# *Babesia gibsoni* Infection in a Cat with Immune-Mediated Haemolytic Anaemia and Thrombocytopenia

**DOI:** 10.3390/ani13132128

**Published:** 2023-06-27

**Authors:** Angel Almendros, Y. R. Choi, Paweł M. Bęczkowski, Kerstin Baiker, Vanessa R. Barrs, Julia A. Beatty

**Affiliations:** Department of Veterinary Clinical Sciences, Jockey Club College of Veterinary Medicine, City University of Hong Kong, Hong Kong SAR, China; yrchoi@cityu.edu.hk (Y.R.C.); pbeczkow@cityu.edu.hk (P.M.B.); kbaiker@cityu.edu.hk (K.B.); vanessa.barrs@cityu.edu.hk (V.R.B.); julia.beatty@cityu.edu.hk (J.A.B.)

**Keywords:** *Babesia gibsoni*, babesiosis, feline, cat, Evan’s syndrome, immune-mediated, anaemia, thrombocytopenia, tick fever

## Abstract

**Simple Summary:**

*Babesia gibsoni* is rarely reported in cats, and its pathogenic potential in this species is unknown. *B. gibsoni* DNA was detected using two pan-*Babesia* PCRs in stored blood from a cat. The cat had died, but retrospective case review identified regenerative anaemia and thrombocytopenia concurrent with *B. gibsoni* detection. Clinical signs resolved on treatment for a suspected immune-mediated aetiology until the cat suffered fatal haemorrhage 6 months later. Samples stored 4 and 6 months from presentation tested negative for *Babesia* spp. This is the first report of *B. gibsoni* detection in a cat to provide clinicopathological information.

**Abstract:**

Tick-borne haemoparasite *Babesia gibsoni* has been detected rarely in cats, in surveys of apparently healthy animals. In stored blood from a 6-year-old male-neutered domestic shorthair cat in Hong Kong, *B. gibsoni* DNA was detected retrospectively using PCR for *Babesia* spp. 18S rRNA and mitochondrial cytochrome B genes, followed by sequencing and basic local alignment search tool (BLAST) analysis. The cat presented with severe haemolytic anaemia and thrombocytopenia. The cat responded to supportive care and glucocorticoids and was clinically normal despite persistent subclinical thrombocytopenia until six months after presentation, when it succumbed to a fatal haemorrhagic episode. Necropsy revealed severe intestinal and pulmonary haemorrhage and hypocellular bone marrow with megakaryocytosis but no other causes of immune-mediated thrombocytopenia (IMTP) or immune-mediated haemolytic anaemia (IMHA). Blood stored on days 158 and 180 tested PCR negative for *Babesia* spp. This report demonstrates that geographic range of *B. gibsoni* detection in cats includes Hong Kong. The exclusion of other causes suggests that *B. gibsoni* might have potentially played a role in triggering immune-mediated disease in this case.

## 1. Introduction

*Babesia* spp. are intraerythrocytic, tick-borne haematozoan parasites that infect a wide range of mammals including cattle, dogs, cats and humans. Babesiosis is a disease complex triggered by infection of some hosts, particularly cattle and dogs, with certain *Babesia* spp.

Understanding the susceptibility of domestic cats to different *Babesia* spp. is emer-ging as molecular diagnosis becomes routine. Most feline babesia infections are subclinical. *Babesia gibsoni* detection was first reported in 5 cats (4%, 5/119) in St Kitts in the Caribbean [1]. More recently, molecular prevalence surveys revealed *B. gibsoni* in one cat in Singapore (0.8%, 1/125) and in twelve cats in mid- and southern China (2%, 12/419) [2,3]. The only report of feline babesia infection in Hong Kong is a case of *B. hongkonensis* described in a free roaming cat (0.3%, 1/300) [4].

*Babesia* infection is common in dogs in Hong Kong with a reported prevalence of up to 40% in the stray population. A recent study identified *B. gibsoni* as the most common *Babesia* sp. infecting dogs in Hong Kong (>90%), followed by *B. vogeli* [5]. Natural transmission in dogs occurs via ixodid ticks, but horizontal transmission through fights, blood transfusions or transplacental transmission have been reported [6]. Similar ways of transmission occur in cats. Although there is no definitive evidence of the regional tick vector species, Muguiro et al. (2023) postulate that *Rhipicephalus sanguineus sensu lato* and *Haemaophysalis longicornis* ticks are likely vectors. Infected dogs are frequently asymptomatic, and the diagnosis of *babesia* infection is serendipitous [7]. The main clinical findings in dogs with babesiosis are regenerative haemolytic anaemia and thrombocytopenia [8]. In dogs, immune-mediated haemolytic anaemia (IMHA) and thrombocytopenia (IMTP) secondary to *babesia* infection are reported [9,10]. NETosis and DIC can additionally contribute towards retention and sequestration of red cells and platelets in spleen and micro-vessels [11]. Differentiation between primary and secondary disease presents a diagnostic conundrum for the clinician, as agglutination, spherocytosis, positive antiglobulins, and thrombocytopenia in the absence of parasitaemia can be present in *B. gibsoni*-infected cases [10]. In cats, on the other hand, primary IMHA or IMTP is rare [12,13]. 

Feline babesiosis is described most commonly in South Africa, with sporadic reports from Europe and Asia [14]. Clinical signs have been described most often in cats infected with *B. felis* [15,16], and occasionally with other species including *B. leo*, *B. microti* and *Babesia* sp. Western Cape [17]. Clinicopathological abnormalities include regenerative anaemia (57%), positive in-saline agglutination tests (16%) and thrombocytopenia (25%) [16]. Hyperbilirubinemia, hyperglobulinaemia and increased liver enzymes are also frequently reported [15]. Pyrexia is not a common feature of babesiosis in cats [15]. 

During a study of residual blood of cats with and without anaemia in Hong Kong, a *B. gibsoni* positive case was identified (City University of Hong Kong Animal Ethics Committee approval number: AN-STA-00000015). A retrospective review of patient data revealed that the cat presented for severe anaemia and concurrent thrombocytopenia, signs which can be consistent with babesiosis. The case is presented herein. 

## 2. Case Description

A 6-year-old neutered male domestic shorthair (DSH) cat was referred to CityU Veterinary Medical Centre (CityU VMC) for severe anaemia (haematocrit [HCT] 9.8%; reference interval [RI] 25–48) and thrombocytopenia (platelet count 0 × 10^9^/L; RI 151–600), confirmed on a blood smear, one day (D) previously (D1) (Table 1). A serum biochemistry profile was unremarkable. Serology for feline leukaemia virus and feline immunodeficiency virus (SNAP FIV/FeLV Combo Test, IDEXX Laboratories Inc., Taipei, Taiwan) were negative.

On presentation at CityU VMC, the owner reported a 2-day history of lethargy and hyporexia, diarrhoea with melena and a single episode of vomiting after drinking water. On physical exam, the cat was quiet, weak and afebrile and had pale mucous membranes, and a grade 3/6 heart systolic murmur was auscultated at the heart base. The body weight was 4.4 kg (body condition score [BCS] 4/9). Ectoparasites were not mentioned in the history or physical examination. The cat lived indoors and had no contact with dogs or other cats.

Results of complete blood count and blood film evaluation on D2 confirmed regenerative anaemia with a haematocrit (HCT) of 10%. Macrocytosis, hypochromasia and moderate reticulocytosis were demonstrated on a blood film evaluation. Total protein (TP) was at the bottom of the normal range at 60 g/L (RI 59–75). Severe thrombocytopenia (24 × 10^9^/L) with abundant macroplatelets, indicative of active thrombopoiesis, was detected (Table 1). There was an increase in band neutrophils with toxic changes consistent with inflammation, and lymphopenia and eosinopenia supported stress or corticosteroid response. Abundant intraerythrocytic inclusions were attributed to basophilic stippling and Howell–Jolly bodies, but no haemoparasites were identified.

Severe thrombocytopenia and anaemia were confirmed, and haemolysis and haemorrhage were supported by clinicopathological findings. Immune-mediated and infectious diseases were investigated further. An in-saline agglutination test was positive macro- and microscopically. Retrovirus serology, feline coronavirus PCR and haemotropic mycoplasmosis PCR testing for *Candidatus M. haemominutum* and for *M. haemofelis* were all negative. Thoracic and abdominal imaging revealed only mild splenomegaly. Immune-mediated haemolytic anaemia and thrombocytopenia of unknown aetiology (Evan’s syndrome) were diagnosed.

Two units (50 mL) of feline packed red blood cells (PRBCs) were administered at 1.6 mL/kg/h given as an intravenous transfusion over 6 h on D2. The HCT improved to 19% 2 h post transfusion. Dexamethasone was administered (1 mg/kg IV) for suspected IMHA and IMTP. Additional medication included maropitant (1 mg/kg slow IV q 24 h).

The cat began eating the following day. The dose of dexamethasone was reduced to 0.3 mg/kg IV q 24 h from D3 to D4. On D4, the platelet count was 139 × 10^9^/L, and the PCV remained stable at 16% with a TP of 62 g/L (Table 1). The cat was eating well and had no further episodes of diarrhoea or vomiting and was discharged from the hospital. 

The case was transferred back to the referring veterinarian for ongoing treatment of concurrent IMHA and IMTP (Evan’s syndrome). The treatment consisted of oral prednisolone (2 mg/kg q 24 h). 

The platelet counts improved and remained mildly below or within the reference range (116–305 × 10^9^/L) for 3 months after the initial episode but decreased (26 × 10^9^/L) further on D105 (Table 1). The HCT was stable during this period, but there was persistent reticulocytosis, suggestive of chronic haemolysis and/or haemorrhage. Despite significant thrombocytopenia, no obvious haemorrhage, petechiae or ecchymoses were observed. The body weight increased to 5.4 kg, and BCS normalised (5/9). Appetite was reported to be very good following the prolonged use of oral prednisolone, continued at 2 mg/kg q 24 h due to persistence of thrombocytopenia. The cat was reported to be active and alert and did not show any of the clinical signs observed previously.

**Figure 1:**
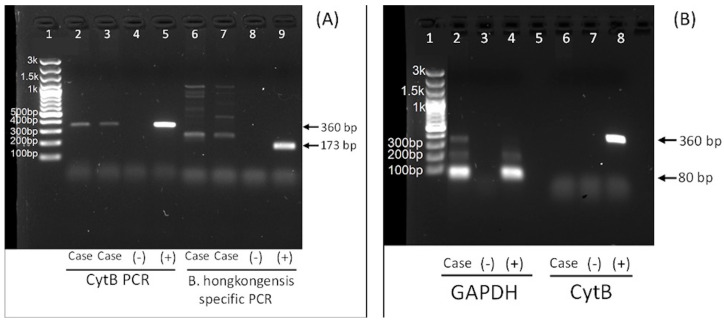
(**A**) PCR result of whole blood DNA collected on Day 1. Lane 1: DNA molecular weight ladder. Lane 2 and 3: duplicate case samples, cytochrome B PCR (“CytB PCR”), positive result (expected product 360 bp). Lane 4: negative control (molecular grade water). Lane 5: positive. Lane 6 and 7: duplicate case samples, *B. hongkongensis* PCR (“*B. hongkongensis* specific PCR”) positive result (expected product 173 bp). Lane 8: negative control (molecular grade water. Lane 9: positive control. (**B**) PCR results from whole blood DNA collected on Day 158. Lane 1: DNA molecular weight ladder. Lane 2: case sample GAPDH PCR (“GAPDH”), positive result (expected product 80 bp). Lane 3: negative control (molecular grade water) for GAPDH PCR. Lane 4: positive control. Lane 5: empty Lane. Lane 6: case sample cytochrome B PCR (“CytB”), negative (expected product 360 bp). Lane 7: negative control (molecular grade water). Lane 8: positive control.

On D105, residual blood that had been collected on D2 was analysed for the presence of *Babesia* spp. as part of an ongoing study. Nucleotide sequence of the 18S rRNA and mitochrondrial cytochrome (Cyt) B genes obtained in this study were consistent with *B. gibsoni* and excluded *B. hongkonensis* or other *Babesia* spp. Retrospectively, *B. gibsoni* DNA was detected in blood taken 24 h after presentation, using *Babesia* spp. 18S rRNA and mitochondrial cytochrome B PCRs, sequencing and BLAST analysis but negative on *B. hongkongensis*-specific PCR (Figure 1). This result was confirmed on repeat DNA extraction and PCR. DNA was extracted from 100 µL of EDTA blood using the DNEasy Blood and Tissue Kit^®^ (Qiagen GmbH, Hilden, Germany) according to the manufacturer’s instructions in a final elution volume of 50 µL and stored at −80 °C until analysis. The integrity of the extracted DNA was confirmed by glyceraldehyde-3-phosphate dehydrogenase (GAPDH) PCR [18]. *Babesia* detection was performed using a pan-*Babesia* PCR targeting Cyt B in piroplasms and a *B. hongkongensis*-specific PCR targeting 18S rRNA as described previously [4]. Each 25 µL reaction contained 1 µL of template DNA, DreamTaq™ Hot Start Green DNA Polymerase (Thermo Fisher Scientific, Graciuno, Vilnius, Lithuania), dNTP (Thermo Fisher Scientific, Graciuno, Vilnius, Lithuania) at a final concentration of 200 μM and a final primer concentration of 500 nM. Molecular grade water was used as negative control. A synthetic DNA construct was used as a positive control with modified bases to allow discrimination between the control sample and wildtype. Primers used, amplicon size and cycling conditions are demonstrated in Table 2.

The target band was cut out and amplicon gel-purified using the PureLink™ Quick Gel Extraction Kit (Thermo Fisher Scientific Hong Kong Limited, Hong Kong). Sanger sequencing was performed (BGI Genomics, Hong Kong), and Geneious software (version 2023.1.1) used to generate the consensus sequence. The sequences of the PCR product from the CytB were compared with known sequences by Basic Local Alignment Search Tool (BLAST) analysis against the NCBI database. The identity was confirmed as *B. gibsoni* with the top hit belonging to *B. gibsoni* isolate (KP666169.1), with E value of 0, query coverage 98% and nucleotide identity 99.44%. Furthermore, the top 50 hits all were *B. gibsoni*, with 95–99% query coverage and percentage identity between 98.84% and 99.44%. CytB sequence assigned Genbank accession number was OR020936. A separate pan-babesia nested PCR was performed to attempt to obtain 18s rRNA sequence [19]. Sequencing of the 18s rRNA PCR product obtained also identified *B. gibsoni*, with the top hit belonging to isolate MN134517.1, with E value of 0, query coverage of 99% and percentage identity of 100%. Overall, all 100 hits belonged to *B. gibsoni*, with query cover of 99% and percentage identity between 99.78% and 100%, assigned 18s rRNA Genbank accession number OQ981666. 

PCR testing for *Babesia* spp. performed subsequently on stored residual blood collected on D158 and D180 was positive for GAPDH but negative for *Babesia* spp. (Figure 1). 

Persistent thrombocytopenia and a regenerative haemogram were present in samples collected from D105 to D158 inclusive (Table 1). The owner reported that the cat was asymptomatic. Body weight increased gradually from 5.4 to 5.7 kg with a BCS of 6/9. On D158, a complete blood count revealed a normal HCT of 38% and persistent thrombocytopenia (platelet count 4 × 10^9^/L (Table 1). Intraerythrocytic inclusions were identified as basophilic stippling and Howell–Jolly bodies, but no haemotropic parasites were identified. Treatment was continued with oral prednisolone (2 mg/kg q 24 h). 

On D180, the cat presented to the emergency service of CityU VMC following an acute onset of weakness, pallor and anorexia after an episode of diarrhoea and melena. The HCT was 9.7%, and the TP was 50 g/L. Shortly after a crossmatched and compatibly typed blood transfusion, the cat experienced a cardiac arrest and did not respond to resuscitation.

**Figure 2:**
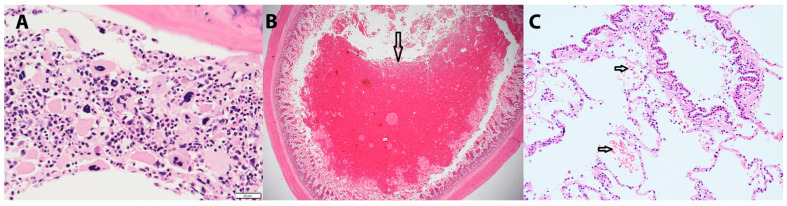
(**A**) Histopathological image from bone marrow showing hypocellularity and megakaryocytic hyperplasia (×40 magnification). (**B**) Histopathological image from mid jejunum showing luminal haemorrhage (black arrow) (×20 magnification). (**C**) Histopathological image from lung tissue with extravasated erythrocytes visible in airways (black arrows) like alveoli and bronchiole (×20 magnification).

A partial post-mortem, consented to on D184, confirmed small intestinal and pulmonary haemorrhage, but no other underlying disease was identified in samples from spleen, jejunum, kidney, liver, pancreas, bone marrow and lung. The bone marrow from a rib showed *post-mortem* related loss of cellular and nuclear details but appeared overall hypocellular with a moderately increased megakaryocyte count evidencing an active platelet regeneration effort due to the thrombocytopenia (Figure 2). The lumen of the small intestine was markedly and diffusely distended by large amounts of extravasated red blood cells with a haemic proportion of leukocytes indicative of acute intestinal haemorrhage (Figure 2). Multiple, small, acute alveolar haemorrhages were present with variable numbers of extravasated red blood cells (Figure 2). Retrospective analysis of blood samples from D158 and D180 as well as DNA extracted from splenic tissue from the *postmortem* on D184 failed to detect *B. gibsoni* on PCR. These findings were suggestive of IMTP causing a fatal haemorrhage and subsequent death. 

## 3. Discussion

This is the first time that *B. gibsoni* has been detected in a cat in Hong Kong. Including this case, *B. gibsoni* infection has been identified by PCR of whole blood DNA from less than 20 cats [1,2,3]. This is also the first time that *B. gibsoni* has been detected in a cat with IMHA and IMTP, although this parasite causes babesiosis in dogs. IMHA and IMTP might have developed in response to *B. gibsoni* infection, which acted as a trigger [20,21]. 

Anaemia, thrombocytopenia, splenomegaly as well as hyporexia, lethargy and weakness are common signs in canine babesiosis. However, to the best of the authors’ knowledge, clinicopathological features associated with this infection in cats have not yet been described. Clinical and laboratory findings in this case were similar to those described for babesiosis due to *B. gibsoni* infection in dogs [7,22]. Pseudothrombocytopenia in vitro in cats is possible and needs to be differentiated when severe low platelet count is present. The initial presentation of IMHA and IMTP might have been triggered by *B. gibsoni* infection, as is reported in dogs, despite later resolution of parasitaemia [9,23]. Although a primary immune-mediated condition is also a possibility in this case, this seems to be rare in cats [12,13]. In dogs, thrombocytopenia can precede parasitaemia [10], and a primary or idiopathic cause of IMHA and IMTP is ruled out or questionable when another mechanism for anaemia is identified [24]. Infiltrative neoplasia such as lymphoma could have explained most clinical signs in this case. However, necropsy and histopathological examination of organs such as spleen, liver, bone marrow and small intestine did not reveal any other underlying causes of secondary IMHA and IMTP. Additionally. although DIC and NET development has been reported in dogs and cannot be excluded in this case, the lack of schystocytes on cytology and the haematological findings with normal white cell count would be less suggestive of their involvement [11].

IMHA resolved following treatment with steroids after initial presentation. Abundant intraerythrocytic inclusions on a blood film were attributed to basophilic stippling and Howell–Jolly bodies that might have masked any merozoites or trophozoites. A low parasite load would have made microscopic identification of any haemoparasites less sensitive. The sensitivity of PCR is superior for diagnostic purposes. The identification of anti-*B. gibsoni* antibodies by indirect fluorescent antibody test (IFAT) in serum might have further supported its involvement in IMHA and IMTP. In this case, however, it is not readily available in Hong Kong and has never been used on cats. IMTP resolved only temporarily but recurred despite the continued use of corticosteroids. IMTP alone has not been reported a major feature in feline babesiosis caused by other *Babesia* spp. [16]. Whether *B. gibsoni* in cats might be a clinical trigger for IMTP is unknown as previous reports were in apparently healthy cats. A persistent secondary and ultimately fatal IMTP, despite molecular clearance of the parasite, is suggested in this case.

Histopathology and clinical pathology were consistent with intestinal and alveolar haemorrhage due to profound thrombocytopenia associated to IMTP. Haemorrhage due to bleeding diathesis associated with severe thrombocytopenia was supported by the low TP when both crises occurred (Table 1). The authors emphasise that due to the retrospective nature of this study, molecular testing and clinical data were only accessible after the cat had passed away. Treatment and management of babesiosis in this case was, therefore, not possible and could not be influenced by the molecular results.

Despite immunosuppression, the failure to amplify the organism on PCR on subsequent occasions when IMTP was severe (D158) and led, eventually, to a fatal haemorrhagic crisis (D180) suggests the molecular clearance of the organism in this case. Such an outcome is uncommon in dogs where recrudescence of the disease due to iatrogenic immunosuppression is more typically expected [8,25]. Babesiosis is unlikely in the absence of anaemia in cats with thrombocytopenia alone [16]. IMTP, either primary and idiopathic or secondary to a previous infection with *B. gibsoni* that acted as a trigger, remained a clinical problem in this cat. Further studies are necessary to understand the course of natural *B. gibsoni* infection in cats and its clinicopathological significance. 

## 4. Conclusions

This is the first case of *B. gibsoni* infection in a cat with IMHA and IMTP. Although the parasitaemia resolved spontaneously, as occurs in dogs, the exclusion of other causes suggests that *B. gibsoni* was a likely potential trigger for immune-mediated disease in this case. A rare primary IMHA and IMTP with a coincidental detection of a non-pathogenic *B. gibsoni* DNA can, however, not be excluded. Studies to determine the prevalence amongst anaemic and non-anaemic cats and amongst the stray and pet cat population will shed more light on the epidemiological aspects of this rare infection. 

## Figures and Tables

**Table 1:** Sequential haematology results.

Test	D1	D2/3/4	D10	D20	D48	D75	D105	D126	D134	D144	D158	D180	Units	Reference Range
**RBC**	**1.81**		**5.08**	7.4	8.2	7.9	8.7	8.6	9.4	8.8	8.56	**2.1**	×10^12^/L	6.9–10.1
HCT/PCV *	**9.8**	10/16/16 *	**26.8**	37.1	38.2	35.9	43.1	42.5.9	41.9	45.2	38.1	**9.7**	%	25–48
**TP**	**57**	60/60/62									**82**	**50**	g/L	59–75
**HGB**	**2.7**		**7.8**	11.0	11.5	11.2	12.8	13.1	13.0	15.8	11.7	**3.4**	g/dL	10.9–15.7
**MCV**	**54.1**		52.8	49.9	46.8	45.2	49.3	49.5	44.6	51.0	44.9	45.7	fl	40.0–52.0
**MCH**	14.9		15.4	14.8	14.0	14.1	14.6	15.2	14.0	17.8	13.6	15.9	pg	13.0–17.0
**MCHC**	**27.6**		29.1	29.6	29.9	31.2	29.7	30.8	**31.0**	35.0	**30.6**	34.8	g/dL	32.0–35.0
**RDW**	**32.6**		**31.2**	27.0	25.3	23.7	24.1	22.4	22.7	21.8			%	15–27
**RETIC**	**99.7**		**69.1**	40.9	**69.9**	**129.6**	**120.8**	**130.5**	**107.9**	**138.4**		**106.0**	K/µL	3.0–50.0
**WBC**	8.2	14/NP/NP	12.6	5.2	4.8	6.4	4.6	5.6	**4.8**	**4.7**	**4.69**	**4.87**	×10^9^/L	5.1–16.2
**NEU**	6.2	**12**/NP/NP	11.7	3.4	3.1	4.7	3.5	4.3	3.8	3.6	3.7	3.4	×10^9^/L	2.3–11.6
**LYM**	1.1	**0.6**/NP/NP	0.9	1.2	**0.8**	0.9	**0.6**	0.8	**0.7**	**0.5**	**0.5**	**0.3**	×10^9^/L	0.9–6.0
**PLT**	**0**	**24** ^^^/NP/**139**	270	305	**159**	**116**	**26**	**23** ^	**16 ^#^^**	**18**	**4 ^#^^**	**0 ^#^**	×10^9^/L	195–624
**MPV**	14.0		16.9	17.2	20.9	19.9	24.1	21.5	20.0	19.6			Fl	11.4–21.6
**PCT**	**0.0**		**0.46**	0.52	**0.08**	**0.23**	**0.05**	**0.05**	**0.02**	**0.04**			%	0.17–0.86

Bold indicates values outside the reference range. * PCV collection, plasma pale yellow. ^#^ Clumps of platelets reported. ^ Macroplatelets reported. D—day, NP—not performed.

**Table 2:** Primers and cycling conditions for PCRs performed in this study.

Primer Set	Name	Use	Sequence	Target Length (bp)	Cycling Condition
CytB [4]	P_cytbF	For	TGTTGCTCCCCAATAACTCATTT	360	Initial denaturation: 95 °C, 3 min Denaturation: 95 °C, 30 s Annealing: 51 °C, 30 s Extension: 72 °C, 1 min, 40 cycles Final extension: 72 °C, 10 min
	P_cytbR	Rev	AGGAATTTAAATTCTAATTGGAATT		
18S rRNA (*B. hongkongensis* specific) [4]	BH_18S565F	For	CGTTTGGGCTTTTAGCTTT	173 bp	Initial denaturation: 95 °C, 3 min Denaturation: 95 °C, 30 s Annealing: 55 °C, 30 s Extension: 72 °C, 1 min, 40 cycles Final extension: 72 °C, 10 min
	BH_18S737R	Rev	TTAACCATTACTAAGGTTCCCA		
GAPDH [18]	GAPDH-For	For	AAGGCTGAGAACGGGAAAC	80 bp	Initial denaturation: 95 °C, 1 min Denaturation: 95 °C, 30 s Annealing: 55 °C, 30 s Extension: 72 °C, 30 s, 35 cycles Final extension: 72 °C, 1 min
	GAPDH-Rev	Rev	CATTTGATGTTGGCGGGATC		

## Data Availability

The original contributions presented in the study are included in the article, and further inquiries can be directed to the corresponding author.
